# M*m*PPOX Inhibits *Mycobacterium tuberculosis* Lipolytic Enzymes Belonging to the Hormone-Sensitive Lipase Family and Alters Mycobacterial Growth

**DOI:** 10.1371/journal.pone.0046493

**Published:** 2012-09-28

**Authors:** Vincent Delorme, Sadia V. Diomandé, Luc Dedieu, Jean-François Cavalier, Frédéric Carrière, Laurent Kremer, Julien Leclaire, Frédéric Fotiadu, Stéphane Canaan

**Affiliations:** 1 CNRS - Aix-Marseille Université - Enzymologie Interfaciale et Physiologie de la Lipolyse - UMR 7282, Marseille, France; 2 CNRS - UMR 7313, Ecole Centrale Marseille - Université Paul Cézanne, Equipe Chirosciences, Marseille, France; 3 Laboratoire de Dynamique des Interactions Membranaires Normales et Pathologiques, Université de Montpellier 2, CNRS, INSERM, UMR 5235, Montpellier, France; 4 INSERM, DIMNP, Montpellier, France; University of Padova, Italy

## Abstract

Lipid metabolism plays an important role during the lifetime of *Mycobacterium tuberculosis*, the causative agent of tuberculosis. Although *M. tuberculosis* possesses numerous lipolytic enzymes, very few have been characterized yet at a biochemical/pharmacological level. This study was devoted to the *M. tuberculosis* lipolytic enzymes belonging to the Hormone-Sensitive Lipase (HSL) family, which encompasses twelve serine hydrolases closely related to the human HSL. Among them, nine were expressed, purified and biochemically characterized using a broad range of substrates. *In vitro* enzymatic inhibition studies using the recombinant HSL proteins, combined with mass spectrometry analyses, revealed the potent inhibitory activity of an oxadiazolone compound, named M*m*PPOX. In addition, we provide evidence that M*m*PPOX alters mycobacterial growth. Overall, these findings suggest that the *M. tuberculosis* HSL family displays important metabolic functions, thus opening the way to further investigations linking the involvement of these enzymes in mycobacterial growth.

## Introduction

According to the World Health Organization (2011; http://www.who.int/tb/en/), tuberculosis remains one of the most threatening and deadly disease in the world, with 8.8 million new infections and 1.5 million deaths in 2010. The emergence of multidrug-resistant (MDR) and extensively drug-resistant (XDR) *Mycobacterium tuberculosis* strains has made the current treatments less efficient. Therefore, the development of new pharmacological strategies to fight this disease are urgently needed [1]. It has been shown that *M. tuberculosis* is able to store triacylglycerols (TAG) as intracellular lipid inclusions (ILI), *in vivo*, during the infection process [2], [3], [4], [5] and *in vitro*, under stress conditions [6], [7]. Additionally, *M. tuberculosis* possesses a vast array of genes coding for enzymes possibly involved in hydrolysis of intra- and/or extracellular lipids, thus allowing the release of fatty acids originating either from the bacteria or from membrane host lipids [8], [9], [10], [11]. Therefore, lipolytic enzymes are thought to play critical roles during the intracellular lifetime of *M. tuberculosis* by participating in the entry into a non-replicating dormant state within host granulomas and/or in dormancy escape, leading to reactivation of the disease.

Lipolytic enzymes are typically divided in four classes, depending on the nature and the specificity of their corresponding substrates: i) carboxylesterases (or esterases) act on small and partially water-soluble carboxylesters; ii) true lipases hydrolyze water-insoluble long-chain carboxylesters, like TAG; iii) phospholipases, acting on phospholipids, are sub-classified into four groups (PLA_1_, PLA_2_, PLC and PLD) with respect to the position of the bond which is cleaved; iv) cutinases constitute a much more versatile family able to degrade carboxylesters of all sorts, including long-chain TAG and phospholipids, as well as cutin [12], [13], [14]. As summarized in Table **S1**, several studies have recently been conducted to identify and characterize several lipolytic enzymes from *M. tuberculosis*. Among them, twelve proteins belonging to the “Lip” family and homologous to the human Hormone Sensitive Lipase (*h*HSL) have been reported [8], [9], [15]. LipY (Rv3097c) [16], [17] was identified as a lipase, while LipC (Rv0220) [18], LipF (Rv3487c) [19], and LipH (Rv1399c) [20] were identified as esterases. Other proteins were only barely characterized [16].

**Figure 1:**
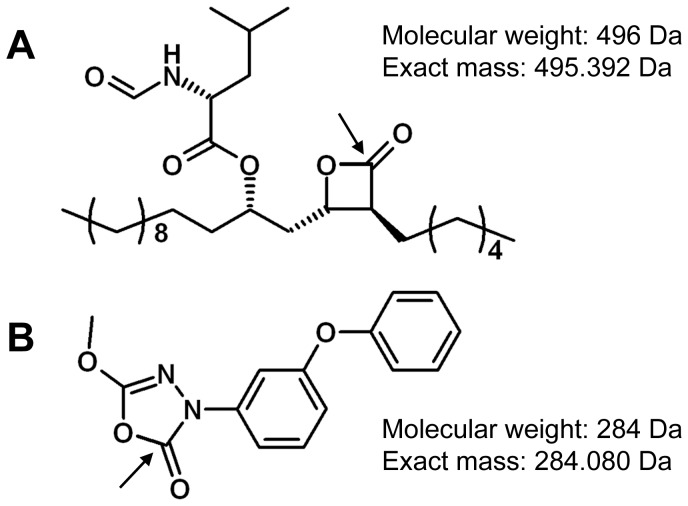
Chemical structure of inhibitors. Chemical structures of **A**, THL and **B**, M*m*PPOX. The proposed mechanism of action involves the opening of the cycle in each molecule. Nucleophilic sites attacked by catalytic serine are indicated by an arrow. Theoretical exact masses were calculated using the online calculator provided by SIS, Inc. (http://www.sisweb.com/referenc/tools/exactmass.htm).

This family of enzymes, referred to as the “Lip-HSL” family, appears particularly interesting regarding the physiological action of *h*HSL, which plays a crucial role in the mobilization of free fatty acid from TAG stored in adipocytes [21], [22]. Therefore, as a first step to decipher the possible link between the “Lip-HSL” family and mycobacterial lipid metabolism, we conducted a biochemical study to characterize substrate specificities of several recombinant Lip-HSL proteins and we investigated their inhibitions using two different compounds. Tetrahydrolipstatin (THL, Figure **1A**), a versatile serine and cysteine hydrolase inhibitor [23], [24] and 5-methoxy-*N*-3-(*meta*-phenoxyphenyl)-1,3,4-oxadiazol-2(3*H*)-one (M*m*PPOX, also known as compound 7600, Figure **1B**), a potent and specific inhibitor of *h*HSL and closely related esterases [25], were used as tools to characterize more precisely Lip-HSL proteins. Effects of these two competitive and covalent inhibitors were also assayed on *M. tuberculosis* and *M. bovis* BCG growth.

## Materials and Methods

### Chemicals

The 5-methoxy-*N*-3-(*meta*-phenoxyphenyl)-1,3,4-oxadiazol-2(3*H*)-one (M*m*PPOX or compound 7600 [25]) was synthesized as previously described [26]. Acetonitrile (ACN), ammonium bicarbonate (NH_4_HCO_3_), 3,5-di-*tert*-4-butylhydroxytoluene (BHT), β-Cyclodextrin (β-CD), dimethyl sulfoxide (DMSO), dithiothreitol (DTT), iodoacetamide (IAA), *N*-lauroylsarcosine, sodium taurodeoxycholate (NaTDC) and tetrahydrolipstatin (THL; Orlistat) were purchased from Sigma-Aldrich-Fluka Chimie (St-Quentin-Fallavier, France). Hygromycin B, isopropyl β-D-1-thiogalactopyranoside (IPTG) and the SDS-PAGE gel stain InstantBlue were from Euromedex (Souffelweyersheim, France). Vinylic esters were purchased from TCI Europe (Zwijndrecht, Belgium).

### Bacterial strains and growth conditions

*Escherichia coli* DH10B cells (Invitrogen) used in cloning experiments were grown at 37°C in Luria Bertani (LB) broth (Invitrogen) or on LB agar plates. Culture media were supplemented with 100 µg/mL ampicillin or 200 µg/mL hygromycin B, when needed. *M. smegmatis* mc^2^155 used for expression experiments was grown at 37°C with shaking (220 rpm) in Middlebrook 7H9 broth (Difco) supplemented with 0.05% Tween-80 (v/v), 0.2% glycerol (v/v), 0.5% bovine serum albumin (BSA) (w/v), 0.2% glucose (w/v) or on Middlebrook 7H11 (Difco) agar plates. Hygromycin B (50 µg/mL) was used for the selection of transformed mycobacteria. *M. bovis* BCG strain Pasteur 1173P_2_ was grown at 37°C in Sauton's medium and *M. tuberculosis* strain mc^2^7000, an unmarked version of mc^2^6030 [27] was grown at 37°C in Sauton's medium supplemented with 24 µg/ml of pantothenic acid.

### Cloning, expression and purification of proteins

The full-length genes encoding *M. tuberculosis* proteins *LipC*, *LipF*, *LipH*, *LipI*, *LipM*, *LipN*, *LipO*, *LipQ*, *LipR*, *LipU*, *LipW*, *LipY* and *Cut6* [16] were amplified by PCR from artificial bacterial chromosomes fragments (Bacmids), full genome, or cosmids of the *M. tuberculosis* H37Rv strain provided by the Pasteur Institute [9], [28] (Table **S1**), using Pfx DNA polymerase (Invitrogen). Cut6 was fused to thioredoxin (TRX) in N-terminal position. For expression in *E. coli*, purified PCR products were cloned into the IPTG-inducible expression vector pDest14 [29], except for Cut6, which was cloned into the petG-20A vector (EMBL). For expression in *M. smegmatis*, purified fragments were ligated into pMyC or pMyNT vectors [30] using NcoI and HindIII restriction sites for expression under the control of the acetamidase promoter. The DNA sequencing of each insert was performed by GATC Biotech (Germany).

For *E. coli*, inductions were performed for 16 hrs with 0.5 mM IPTG when cultures reached the mid-log exponential growth phase and cells were harvested by centrifugation. Soluble proteins (LipN and Cut6) were purified as follows: cells were resuspended in ice-cold lysis buffer (50 mM Tris-HCl, pH 8.0, 150 mM NaCl, 0.1% Triton X-100, and 0.25 mg/mL lysozyme, 30 mL/L of initial culture with a final OD_600 nm_ of 4). Supernatants obtained after centrifugation were loaded (1 mL/min) onto a Ni^2+^-NTA resin (1 mL/5 mg of recombinant protein) previously equilibrated with buffer A (Table **S2**), using an FPLC chromatography system (Amersham Biosciences). The column was washed with 5 column volumes of buffer A then washed using five column volumes of 5% and 10% of buffer B (buffer A+500 mM imidazole). Enzymes were eluted with 50% of buffer B and fractions of eluted peaks containing purified recombinant proteins were analyzed by SDS-PAGE. For Cut6, the TRX fusion protein was removed by proteolytic cleavage using the tobacco etch virus (TEV) protease. Insoluble proteins expressed as inclusion bodies (LipH and LipI) were recovered in the pellet, purified and refolded as previously described [20] using the appropriate buffer (Table **S2**).

Preparation of *M. smegmatis* competent cells and electroporation procedures were performed as described previously [30]. Cells were grown in 7H9 complete medium containing 50 µg/mL hygromycin B at 37°C with shaking until an OD_600_ value of 3 was reached. Expression of recombinant proteins was induced for 16 hrs by adding acetamide to a final concentration of 0.2% (w/v). Cells were harvested, resuspended in buffer A containing 1% *N*-lauroylsarcosine (Table **1**) and processed for purification as previously described [30].

**Table 1:** Substrate specificity of recombinant Lip-HSL proteins.

	Substrate chain length/specific activities^a^ (U/mg)					
	*p*NP esters^b^		Vinyl esters^c^		TAG^d^	
Protein	Best	Up to	Best	Up to	Best	Up to
LipC [18]	C4/0.12	C10/0.02	*n.d*	*n.d*	*n.d*	*n.d*
LipF	C4/0.18	C10/0.02	*n.d*	*n.d*	*n.d*	*n.d*
LipH [20]	C4/13.5	ND	C3/1600	C4/1100	C3/1350	C4/450
LipI	C4/15.7	ND	C4/73	C4/73	C3/30	C4/27
LipN	C4/7.2	C14/0.04	C4/1390	C4/1390	C3/350	C4/240
LipR	C8/0.53	C12/0.27	*n.d*	*n.d*	*n.d*	*n.d*
LipU	C8/0.48	C14/0.04	*n.d*	*n.d*	*n.d*	*n.d*
LipW	C4/1600	C14/0.04	*n.d*	*n.d*	*n.d*	*n.d*
LipY	C4/46.3	C14/5.2	C4/300	C8/35	C4/208	C18:3/4.0

a All activities were performed beyond the substrate solubility limit (except for pomegranate oil, which was directly coated on the plate) and 1 unit (U) corresponds to 1 µmol of fatty acid released per min.

b Chain lengths tested include C4, C5, C8, C10, C12 and C14.

c Chain lengths tested include C4, C6 and C8.

d Chain lengths tested include C3, C4, C8, C18:1 (olive oil) and C18:3 (pomegranate oil).

ND not determined.

*n.d* not detected.

Purified proteins were concentrated between 0.5 and 3 mg/mL, and stored at −80°C. Theoretical physical properties (molecular mass, extinction coefficient at 280 nm and isoelectric point, including the His_6_-tag) of all proteins were obtained from the ProtParam tool (http://ca.expasy.org/tools/protparam.html). Data are summarized in Table **S1**.

### Enzymatic activity assays on *p*-nitrophenyl (*p*NP) esters

Activities were measured using *p*NP esters of butyrate (*p*NPC4), valerate (*p*NPC5), caprylate (*p*NPC8), caprate (*p*NPC10), laurate (*p*NPC12) and myristate (*p*NPC14). Release of *p*NP was monitored at 410 nm and pH 7.5 using a 96-well plate spectrophotometer (PowerWave™, Bio-Tek Instruments) and quantified using a *p*NP calibration curve (10 µM to 0.5 mM) with apparent ε_(λ = 410 nm)_ = 8.4 mM^−1^ and 6.0 mM^−1^ when using 0.5% Triton X-100 (w/v). Enzymatic reactions were performed at 37°C for at least 10 min in a 100 mM Tris-HCl buffer (pH 7.5) containing 100 mM NaCl, various amounts of enzyme (5–200 µg) and 2 mM of substrate initially solubilized in ACN (100 mM). Final volumes were fixed to 200 µL in each microtiter well. In the case of *p*NPC8 and longer chain lengths, substrates were first solubilized in buffer containing 0.5% Triton X-100 (w/v) by sonication in a waterbath for 1 min.

Activities were expressed in international units (U), corresponding to 1 µmol of *p*NP released per min. Specific activities were expressed as U/mg of pure enzyme. Negative controls included denaturated enzymes (10 min boiling) and protein filtrates (obtained during concentration steps). All experiments were performed at least in duplicate.

### pH-stat assays

Enzymatic hydrolysis of emulsions of various esters, namely vinyl butyrate (VC4), hexanoate (VC6), and caprylate (VC8) as well as tripropionin (TC3), tributyrin (TC4), tricaprylin (TC8) and triolein (TC18:1) were monitored titrimetrically for 10 min at 37°C using a pH-stat (Metrohm 718 STAT Titrino; Metrohm Ltd., Herisau, Switzerland). Substrate concentrations and solubility limits were taken as previously published [31]. Assays were performed in 2.5 mM Tris-HCl buffer (pH 8) containing 150 mM NaCl (pH 7.5 and 300 mM for LipY). Various concentrations of bile salts (NaTDC), ranging from 0.25 to 4 mM, were assayed to improve substrate emulsification. Free fatty acids released were automatically titrated with 0.1 M NaOH to maintain a fixed end-point pH value of 8.0.

### Lipase activity assays on TAG from Pomegranate oil

Corning UV 96-well microplates were coated as previously described [32], [33] using TAG from Pomegranate oil, containing up to 80% punicic acid (C18:3) equally present at the *sn*-1, *sn*-2 and *sn*-3 positions of the glycerol backbone. The lipase activity was measured at 37°C in 10 mM Tris-HCl buffer (pH 7.5) containing 150 mM NaCl, 6 mM CaCl_2_, 1 mM EDTA, 0.001% (w/v) 3,5-di-*tert*-4-butylhydroxytoluene (BHT) and 3 mg/mL β-Cyclodextrine (β-CD). The formation of the β-CD/free punicic acid complex was continuously monitored at 275 nm for 60 min.

### Inhibition by M*m*PPOX and THL

Inhibition experiments were carried out using a classic lipase-inhibitor pre-incubation method, as previously described by Ransac *et al.* [34]. Briefly, stock solutions (from 10 µM to 100 mM) of M*m*PPOX or THL were prepared in ACN or DMSO, respectively. Each enzyme was further pre-incubated at 25°C with each inhibitor, at various inhibitor molar excess (*x*_I_), ranging from 0.5 to 500 related to 1 mol of enzyme. Higher concentrations of inhibitor were not assayed, as these two compounds tend to precipitate in aqueous solution. Control experiments were performed with the same volume of solvent, without inhibitor. Residual activities were assayed at different incubation times using the pH-stat technique or colorimetric assays using *p*NP esters as described above. At the end of the 30 min incubation period, samples were filtered and aliquots were loaded on SDS-PAGE gel to confirm the recovery of all the protein in the supernatant.

As IC_50_ are not relevant values when assaying insoluble and competitive inhibitors, like THL and M*m*PPOX, we rather used *x*_I50_ and t_1/2_ values [35]. *x*_I50_ were defined as the inhibitor molar excess leading to 50% of lipase residual activities after 10 min incubation time. Thereby, a *x*_I50_ value of 0.5 is synonymous with the rapid formation of a 1∶1 stoichiometric lipase-inhibitor adduct, and is the highest level of inhibitory activity that can be achieved here. Half inactivation times (t_1/2_) were defined as the time needed to reach 50% lipase residual activity at a given *x*_I_ value. As inhibitors and substrates used in this study are mostly insoluble, the Michaelis-Menten-Henri model no longer applies [36] and calculated *K*_m_ and *K_i_* are only apparent values, arising from multiple and complex partitioning equilibria [37]. Results are expressed as mean values of at least two independent assays (CV%<5.0%).

### Protein digestion using trypsin or chymotrypsin

In-gel digestion of proteins were performed with sequencing grade trypsin or chymotrypsin (Sigma-Aldrich and ProteaBio Europe, respectively), following the manufacturer's instructions. Briefly, protein bands were excised from the 12% SDS-PAGE gel, cut into small pieces, washed (50% ACN in 100 mM NH_4_HCO_3_) and successively treated with 10 mM DTT in 100 mM NH_4_HCO_3_ buffer (pH 8.0) for 45 min at 56°C and 55 mM IAA in 100 mM NH_4_HCO_3_ buffer for 30 min at 25°C in the dark. Supernatants were discarded and gel pieces were washed twice (50% ACN in 100 mM NH_4_HCO_3_) before being dried. Trypsin or chymotrypsin (10 µg/mL, in a 25 mM NH_4_HCO_3_ buffer) were added and reactions were performed overnight at 37°C. Peptides were extracted several times with 0.1% trifluoroacetic acid (TFA) in water and ACN (50∶50, v/v). Fractions were pooled together, concentrated and desalted prior to analysis.

### Matrix-Assisted Laser Desorption Ionization Time-of-Flight (MALDI-TOF) mass spectrometry analysis

A saturated solution of α-cyano-4-hydroxycinnamic (Sigma-Aldrich) acid in acidified water (0.1% TFA) and ACN (30∶70, v/v) was used as a matrix for peptide analysis. Equal volume of peptides and matrix were mixed together and spotted on the target. For global mass analyses, 20–100 pmol of desalted protein solutions were mixed with 1 µL of a saturated solution of sinapinic acid (Sigma-Aldrich) in acidified water (0.1% TFA) and ACN (60∶40, v/v) and spotted on the target. Samples were allowed to air dry at 25°C. MALDI-TOF analyses were performed on a Microflex II mass spectrometer (Bruker, Daltonik, Germany). Mass spectra were acquired in positive ion mode, using the FlexAnalysis software (Bruker, Daltonik, Germany). Spectra were externally calibrated using a solution of proteins or peptides standards (Sigma-Aldrich). Spectra were further internally recalibrated using characteristic signals of protease. Proteins identifications were carried out using MASCOT (http://www.matrixscience.com/) search engine against NCBI database. Theoretical and experimental peptides masses were obtained using the BioTools software (Bruker, Daltonik, Germany).

### Drug susceptibility testing

The susceptibility of *M. tuberculosis* and *M. bovis* BCG to THL and M*m*PPOX were determined as reported previously [38]. In brief, Middlebrook 7H10 solid medium containing oleic-albumin-dextrose-catalase enrichment (OADC) and 24 µg/ml of pantothenic acid (for *M. tuberculosis*), was supplemented with increasing drug concentrations. Serial 10-fold dilutions of each actively growing culture were plated and incubated at 37°C for two to three weeks. The minimal inhibitory concentration (MIC) was defined as the minimum concentration required to inhibit 99% of the growth.

## Results

### Targets selection

Mining the *M. tuberculosis* H37Rv genome database [8], [9] revealed the presence of 36 genes encoding putative lipolytic enzymes (α/β hydrolase fold). These genes were classified in different categories depending on their sequence homology with previously biochemically characterized lipases and esterases (Table **S3**). Briefly, one gene was found to be homologous to a *Candida parapsilosis* lipase, one to the human bile salt-dependent lipase (BSSL), 12 to the *h*HSL [16], [17], [19], [20], 7 to the *Fusarium solani* cutinase (*Fs* cutinase) [39], [40], [41], [42], [43], 4 to monoacylglycerol lipases [44], [45] and 4 to phospholipases C (PLC) [30]. One unusual enzyme (Rv1683), possessing an acyltransferase domain in C-terminal region and a lipase domain, homologous to human gastric lipase, in N-terminal region, was recently characterized [46]. Six additional enzymes with no identity with known lipolytic enzymes, but bearing the minimal consensus sequence for serine hydrolase (GX**S**XG, where G is a glycine, X could be any residue distinct from proline and **S** is the catalytic serine residue [47]), were also considered as putative lipolytic enzymes.

The use of an inhibitor specifically targeting members of a given family of proteins may be particularly helpful to address the contribution and requirements of this family in the physiology and growth of mycobacteria. Therefore, we reasoned that the availability of M*m*PPOX, a known *h*HSL specific inhibitor [25], may provide new insight regarding the role of the Lip-HSL family in *M. tuberculosis* and *M. bovis* BCG. To this aim, all genes encoding putative Lip-HSL members were amplified and cloned into *E. coli* or *M. smegmatis* expression vectors for large scale production of recombinant proteins. Cut6, belonging to the cutinase family [39], [48] and presumably essential for the mycobacterial growth [42], [49], [50], was also included in this study. Indeed, this enzyme possesses a catalytic serine and displays carboxylesterase, phospholipase as well as thioestererase activities, and thought to be a valuable candidate to assess in order to determine M*m*PPOX and THL selectivity and efficiency.

### Expression and purification of recombinant proteins

Expression of recombinant Lip-HSL proteins in *E. coli* led to insoluble proteins, with the exception of LipN. In this case, 10–15 mg of purified protein were typically recovered from 1 liter or culture. All other proteins were recovered as inclusion bodies with typical yields ranging from 50 to 100 mg/L of culture, prompting us to choose a method based on solubilization of inclusion bodies to renaturate the recombinant proteins [51]. Protein solutions were concentrated up to 1–2 mg/mL and traces of urea and imidazole were removed by gel filtration or dialysis. However, only LipH and LipI were recovered as active enzymes, with typical yields of 5–10 mg/L. When renaturation of proteins failed, *M. smegmatis* was used as a surrogate expression host. Six out of nine HSL proteins were then obtained in high yields: for LipC [18], up to 120 mg of pure and active recombinant enzyme were recovered from 1 liter of culture, whereas up to 60 mg/liter of culture were obtained for LipM, LipO, LipQ, LipW and LipY [52]. LipF, LipR and LipU were moderately expressed (5–10 mg/L of culture), despite the addition of higher concentrations of inducer (acetamide up to 2% w/v) in the culture medium.

**Figure 2:**
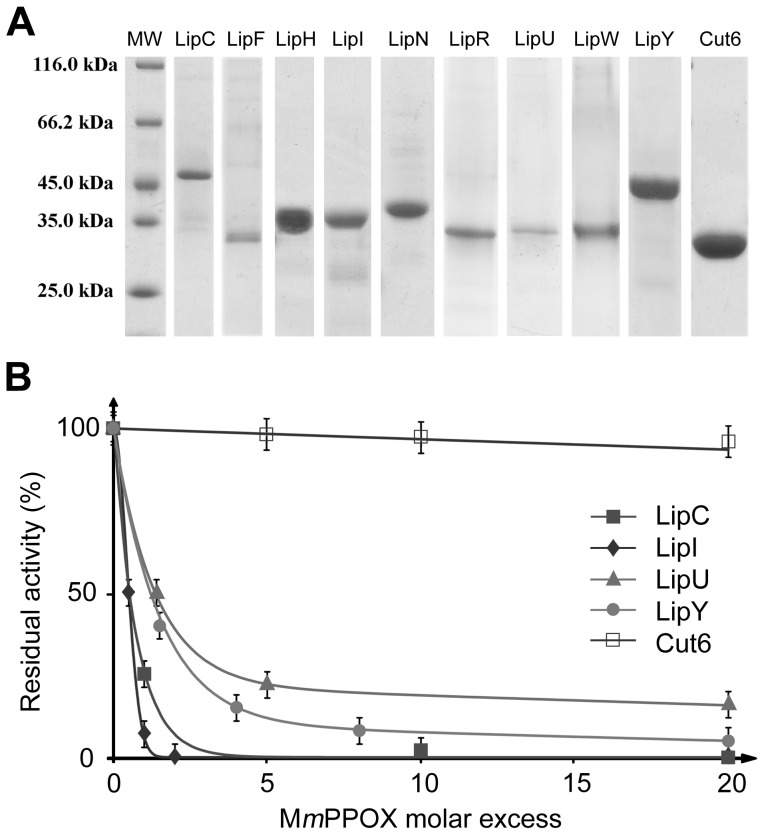
Inhibition of Lip-HSL proteins by M*m*PPOX. **A**, SDS-PAGE profile of the 9 Lip-HSL proteins used in this study, following purification using Ni^2+^-NTA resin. Quantity loaded: Molecular Weight (MW), 2 µg; LipC (46 kDa), 2 µg; LipF (31 kDa), 1 µg; LipH (36 kDa), 5 µg; LipI (36 kDa), 6 µg; LipN (42 kDa), 5 µg; LipR (34 kDa), 3 µg; LipU (33 kDa), 1 µg; LipW (34 kDa), 3 µg; LipY (47 kDa), 10 µg; Cut6 (31 kDa), 9 µg. **B**, Residual activities of LipC, LipI, LipU, LipY and Cut6 after 10 min incubation with M*m*PPOX at various molar excess (*x*_I_). Residual activities were measured spectrophotometrically using *p*NPC4 as substrate. *x*_I50_ values were defined as the inhibitor molar excess leading to 50% enzymes residual activities.

Purification procedures using Ni^2+^-NTA resin usually provided proteins with purities of 90% (Figure **2A**), which were substantially improved by an additional gel filtration step, leading >95% purity. Due to aggregation, LipM, LipO and LipQ were repeatedly found to be inactive after this purification step and, therefore, not further characterized in this study.

### Lipolytic activity determination

As computational analyses do not allow one to clearly discriminate esterases from true lipases within the α/β hydrolase fold family, *in vitro* assays using a vast array of substrates (including short-, medium- and long-chain fatty acid esters) were performed for each recombinant protein. These data will ensure the right classification as carboxylesterase or true lipase and, *in fine*, will be very helpful to better understand the physiological functions of Lip-HSL proteins.

The synthetic substrate *p*NPC4 was used to check for enzyme activity and stability during final refolding and concentration steps. Other substrates with longer acyl chain lengths, such as synthetic vinyl esters and natural TAG, were used to investigate substrate specificity. Finally, lipase activity was monitored spectrophotometrically thanks to a sensitive method based on coated TAG from pomegranate oil (C18:3) as a substrate [32]. Results are summarized in Table **1**. LipC and LipW exhibited activities on *p*NP esters with a preference for short chain substrate (123±15 and 1600±75 mU/mg with *p*NPC4, respectively). LipU and LipR showed substantial activity with all *p*NP esters tested, but exhibited a preference for medium-chain substrates (485±10 and 425±18 mU/mg with *p*NPC8, respectively). None of these four enzymes exhibited activity on emulsified vinyl esters or TAG (including pomegranate oil), indicating that these proteins were more likely acting as esterases rather than lipases. LipI, LipN and LipY were found to be preferentially active on short-chain *p*NP and vinyl esters (73±6, 1390±110 and 300±5 U/mg with vinyl butyrate, respectively). Activity was also detected with short-chain emulsified TAG: tripropionin (30±3, 350±15 and 75±6 U/mg, respectively) and tributyrin (27±3, 240±15 and 208±14 U/mg, respectively). As previously reported [19], [20], LipH and LipF were found preferentially active on short-chain substrates (1350±100 U/mg with tripropionin and 176±20 mU/mg with *p*NPC4, respectively). These data indicate that the above-mentioned enzymes may also be considered as carboxylesterases. LipY was the only enzyme with activity on long-chain TAG (4020±160 mU/mg with pomegranate oil), thus presenting a true lipase activity. Thioesterase, phospholipase or protease activities were not assayed in this study.

### *In vitro* inhibition studies

Each enzyme was assayed individually in the presence of M*m*PPOX. To allow comparison between inhibition data, incubation and residual activity determination were performed using the same experimental procedure. Since all recombinant enzymes exhibited an activity with the short-chain soluble substrate *p*NPC4, this substrate was chosen to assay the residual activity of all enzymes. As depicted in Table **2**, at inhibitor-to-enzyme molar excess of 20 (*x*_I_ = 20), all Lip-HSL enzymes tested were found to be strongly inhibited (less than 15% remaining activities after a 10 min incubation period) with half inactivation times (t_1/2_) shorter than 1 min. In particular, high inhibition rates (t_1/2_<1 min and remaining activities <10%) were obtained with LipC, LipF, LipI and LipN, even at near stoichiometric proportions of inhibitor (*x*_I_<2). A slight, but reproducible, activity restoration occurred with LipF, lipR, LipU and LipW: residual activity increased by 4–8% after 30 min incubation as compared to activity measured after 10 min. These results suggest that the inhibition by M*m*PPOX is partly reversible, in agreement with a recent study [53]. By contrast, Cut6, a non Lip-HSL protein possessing a catalytic serine, retained full activity in the presence of M*m*PPOX at *x*_I_ = 20.

**Table 2:** Inhibition studies using M*m*PPOX.

			Residual activity (%)	
Protein	Activity on *p*NPC4 (U/mg)	*x*_I_	10 min	30 min
LipC	0.12±0.02	5	0	0
		2	7	5
LipF	0.18±0.02	2	0	0
		1	7	11
LipH	13.5±1.5	10	0	0
		5	9	9
LipI	15.7±3.2	2	0	0
		1	7	6
LipN	7.2±0.7	5	0	0
		2	6	6
LipR	0.53±0.02	20	7	15
LipU	0.49±0.02	20	10	17
LipW	1.6±0.1	20	6	13
LipY	33.3±1.8	20	5	5
Cut6	0.26±0.02	20	100	100

Inhibition constants (*x*_I50_ and apparent *K*_i_) were determined for several representative Lip-HSL members, namely, LipU, LipI and LipC, expressing respectively, high, intermediate and low residual activity after 30 min of incubation with M*m*PPOX. *x*_I50_ and apparent *K*_i_ were also determined for LipY, the only Lip-HSL protein with a true lipase activity and the non-HSL protein Cut6 (Figure **2A**). Identical experimental conditions were applied to generate relevant and comparable values. Data, summarized in Table **3** and Figure **2B**, clearly point out to the potent inhibition activity of M*m*PPOX towards Lip-HSL proteins. Indeed, *x*_I50_ values for LipI and LipC were found to be 0.5, while LipU and lipY displayed *x*_I50_ values<1.5, leading to apparent *K*_i_<10 µM. In contrast, a high *x*_I50_ value (227.9) was determined for Cut6, in line with an apparent *K*_i_>1 mM (*K*_i_ = 6.4 mM), about 1000 times higher than those of Lip-HSL proteins. The same kinetic constants were measured for THL, under the same experimental conditions (Table **3**). This inhibitor was found to be much more versatile than M*m*PPOX, as depicted by the strong discrepancy within *x*_I50_ values. This is exemplified by the capacity of THL to strongly inhibit Cut6 (*x*_I50_<10), as previously described [41], [42], compared to its lack of activity against LipC or LipU even at high molar excess (*x*_I_>500). Regarding LipY, both inhibitors showed similar efficiency with low *x*_I50_ (<1.5) and apparent *K*_i_ (<10 µM). In addition, activity restoration was also observed with THL (data not shown). The reversibility of the inhibition by THL in the presence of substrates was already reported [54].

**Table 3:** Inhibition constants of M*m*PPOX and THL.

		M*m*PPOX		THL		
Protein	*K*_m_ a (mM)	*x*_I50_	Apparent *K*_i_ (µM)	*x*_I50_	Apparent *K*_i_ (µM)	*x*_I50_ ratio^b^
LipC	0.18	0.5	1.0	>500	>10^3^	>10^3^
LipI	0.13	0.5	0.1	65.9	13.1	131.8
LipU	0.62	1.5	5.1	>500	>10^3^	>333
LipY	1.39	1.2	7.7	0.5	3.2	0.4
Cut6	3.26	227.9	>10^3^	9.7	141.9	<0.1

a Apparent *K*_m_ on *p*NPC4.

b Calculated as: *x*_I50_ (THL)/*x*_I50_ (M*m*PPOX).

### Mass spectrometry analyses

**Figure 3:**
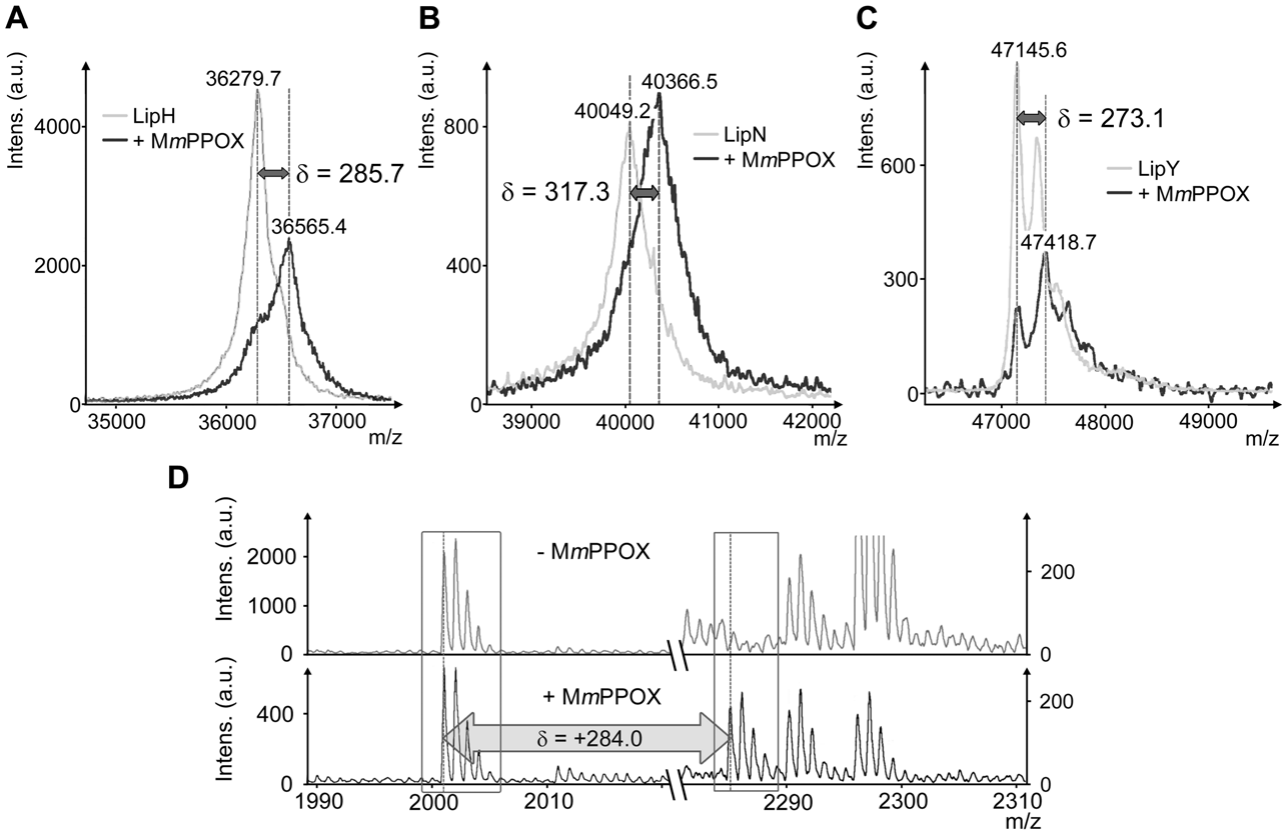
Protein-inhibitor adducts studies using mass spectrometry. Global mass modifications of **A**, LipH; **B**, LipN and **C**, LipY after 30 min incubation with M*m*PPOX at a molar excess of 20 (*x*_I_ = 20). **D**, PMF spectra of LipN before (top) and after (bottom) 30 min incubation with M*m*PPOX at a molar excess of 20 (*x*_I_ = 20). Only two parts of interest are shown: left part, area for a catalytic peptide, containing the unmodified catalytic Serine (*m/z*, 2001.0); right part, area expected for the appearance of a new signal for the catalytic peptide following reaction of the catalytic Serine with M*m*PPOX (*m/z*, 2285.0; δ = +284.0). Note that, for the sake of clarity, different vertical scales were chosen for the left parts, but identical vertical scales were chosen for the right parts.

MALDI-TOF mass spectrometry was further used to appreciate the covalent nature of the inhibition. At *x*_I_ = 20, mass increments of +286, +317 and +273 Da were observed within global masses of LipH, LipN and LipY, respectively (Figure **3A–C**). These data are in agreement with the formation of a covalent enzyme-inhibitor adduct, as the reaction between M*m*PPOX and the catalytic Serine is expected to yield a mass increase of +284 Da. Concerning LipI, protein degradation occurred during signal acquisition, preventing the detection of any mass increase (data not shown). Regarding LipC, LipF, LipR, LipU or LipW, masses were found unchanged as compared to control experiments, even by increasing *x*_I_ to 40 (data not shown). This may be linked to the reversibility of the inhibition process and the lability of the enzyme-inhibitor adduct, as reported previously.

Peptide mass fingerprinting (PMF) is a useful tool to identify proteins and probe mass modifications within specific peptides [55]. This method was applied here i) to confirm the identity of the recombinant protein obtained after purification steps and ii) to confirm the covalent binding of the inhibitor to the catalytic Serine residue, thus excluding any non-covalent inhibition (*i.e.* fixation of the inhibitor near the active site, blocking its access). Proteins were first in-gel digested using trypsin or chymotrypsin to generate peptides mixtures (0.6–4 kDa), which were further analyzed using MALDI-TOF mass spectrometry. Peptides harboring the catalytic Serine residue (referred to as catalytic peptides) were finally identified within the spectrum. It is noteworthy that the oxidation state of Methionines present in these peptides could be different (+16.0 Da per oxidized Methionine) and that miss-cleavages can occur during proteolysis (usually, up to 1 using trypsin and up to 3 using chymotrypsin). Consequently, several catalytic peptides can be identified within the same spectrum. For LipC, however, no catalytic peptides were detected, regardless the protease used (Table **S4**). Other proteins, yielding at least one or two catalytic peptides, were incubated with M*m*PPOX at a *x*_I_ value of 20. After digestion, the peptide mixture was expected to display additional MALDI-TOF signals, corresponding to catalytic peptides shifted by a mass increment of +284.1 Da after reaction with M*m*PPOX. As anticipated, a modified catalytic peptide (+284.4 Da) was detected in LipI (Table **S4**). LipN exhibited a modification of +284.0 Da within the single peptide containing its catalytic Serine residue (see Table **S4** and Figure **3D**). Similarly, two catalytic peptides were identified in LipH (differing by the oxidation state of a Methionine, +16.0 Da), each presenting molecular weight shifts of +284.1 Da after incubation with M*m*PPOX. Overall, these results corroborate and support the hypothesis of a covalent reaction of the inhibitor with the catalytic Serine. It is noteworthy that, in each case, unmodified peptides were still present in spectra with lower intensities, as illustrated in Figure **3D**. Regarding LipF, LipR, LipU, LipW and LipY, no modifications were observed, even when using *x*_I_ of 40. The multiple steps required for sample preparation, involving high temperatures and detergent concentrations, combined with the reversibility of the inhibition by M*m*PPOX, may, at least partly, contribute to the absence of the desired modifications.

### Effect of M*m*PPOX on mycobacterial growth

**Figure 4:**
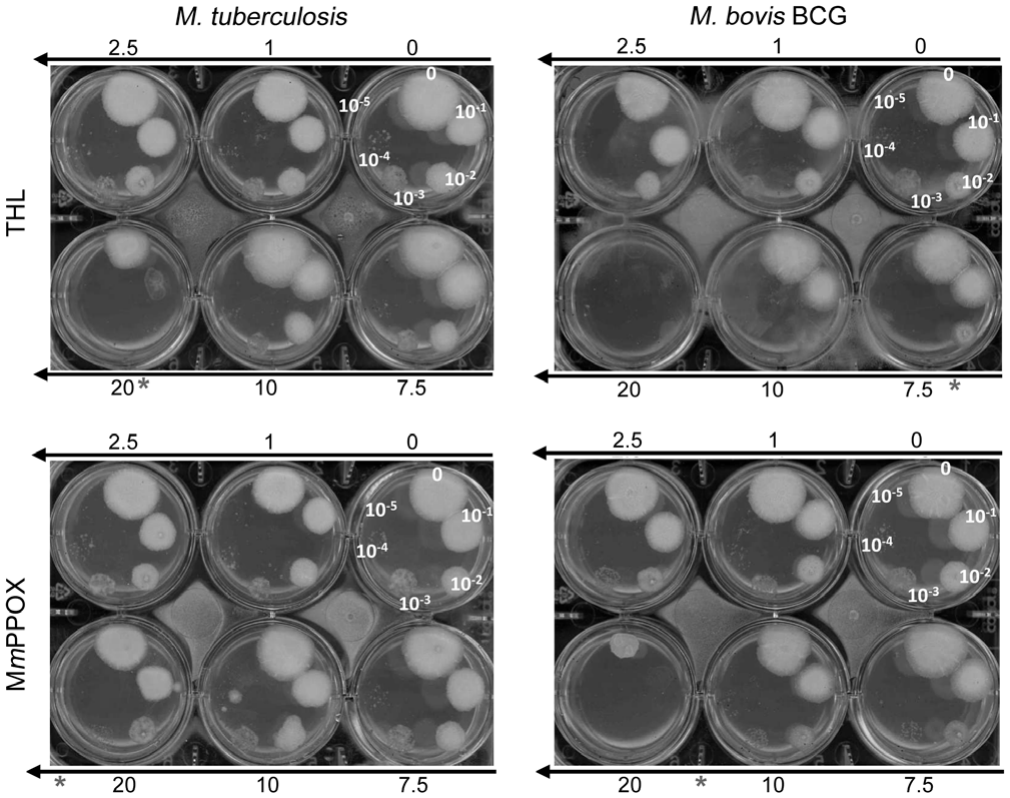
Antimycobacterial activity of M*m*PPOX and THL. Susceptibilities of *M. tuberculosis* were determined on solid medium with increasing inhibitor concentrations (1–20 µg/mL). Serial 10-fold dilutions (indicated on plates) of an actively growing culture were plated and incubated at 37°C for 2–3 weeks. MIC were defined as the minimum concentration required to inhibit 99% of the growth. MIC against *M. tuberculosis* and *M. bovis* BCG are indicated with a star and estimated to be 20 and 7.5 µg/ml for THL and >20 (around 25) and between 10–20 µg/mL for M*m*PPOX, respectively.

Previous observations indicated that THL inhibited the growth of *M. tuberculosis* [49], [56] and the catabolism of intracellular lipidic inclusion (ILI) in *M. smegmatis*, reducing its growth [57]. These results suggest that lipolytic enzymes could be regarded as potent targets for future drug development. In this context, we evaluated the effect of M*m*PPOX on *M. tuberculosis* and *M. bovis* BCG to compare its ability to inhibit bacterial growth with respect to THL. As shown in Figure **4**, M*m*PPOX was also found to inhibit the growth of *M. tuberculosis* and *M. bovis* BCG with MIC values of about 25 and between 10–20 µg/mL, respectively. These two values were however slightly higher than those found for THL (20 and 7.5 µg/mL, respectively). Keeping in mind the strong affinity of M*m*PPOX toward the Lip-HSL family, it can be inferred that inhibition of these enzymes is likely to occur in mycobacteria. Further experiments are, however, required to confirm the hypothesis that inhibition of the Lip-HSL family members by M*m*PPOX could is responsible for the mycobacterial growth defect observed here.

## Discussion

We report here purification, characterization and *in vitro* inhibition studies of nine out of twelve *M. tuberculosis* Lip-HSL candidates, thus providing a more detailed portrait of this important family of enzymes. This first study reported that this family comprises only esterases, with the exception of LipY, identified as a true lipase able to hydrolyze insoluble long-chain TAG, as previously mentioned [16], [17]. Based on this result, one can propose LipY to participate in the degradation of intra- or extracellular TAG, thus releasing fatty acids able to fuel the glyoxylate cycle. However, other Lip-HSL members may take part in metabolic processes or pathways involving short-chain substrates, such as signalling, membrane support and regulation. Whether these enzymes are expressing additional phospholipase, thioesterase or protease activities is currently being examined and should provide valuable information with respect to their physiological role *in vivo*.

All purified Lip-HSL proteins were, *in vitro*, strongly inhibited by M*m*PPOX at low molar excess (*x*_I_), ranging from 1 to 20 (Table **2**). Kinetic constants of inhibition, at least for several Lip-HSL representative members, highlighted their strong affinity for M*m*PPOX, with an apparent *K*_i_ value as low as 0.1 µM for LipI (Table **3**). When investigating the mode of action of this inhibitor, mass spectrometry analyses supported the fact that the catalytic Serine was modified, subsequently confirming the assumption that M*m*PPOX acts as a competitive and covalent inhibitor able to react with the catalytic Serine. These results are consistent with previous studies on *h*HSL-related bacterial esterases from *Alicyclobacillus acidocaldarius* and *Archaeoglobus fulgidus* [25].

Another aspect revealed by this study concerns the reversibility of the inhibition mechanism. When assaying LipC, LipF, LipR, LipU or LipW, lipolytic activity was partially recovered after 30 min of incubation with M*m*PPOX. This notion of reversibility was supported by mass spectrometry analyses, since mass increments were not detected for any of these five enzymes. Regarding LipY, mass measurements revealed that about 25% of the native form of the enzyme was present simultaneously with the inhibited form (Figure **3C**), indicating a slow reversibility. This fact was subsequently confirmed by PMF experiments, as modified catalytic peptides were not observed at all. Activity restoration was reported recently in the case of *h*HSL, for which the enzyme-M*m*PPOX adduct could be hydrolyzed with time, releasing an oxadiazolone decomposition product and the active form of the enzyme [53]. In these conditions, such an inhibitor can be considered as a long-life substrate rather than a true inhibitor [58]. It is difficult to estimate to which extent this characteristic could affect the efficiency of the inhibitor *in vivo*.

A close inspection of the *in vitro* inhibition kinetics indicated that, unlike THL, M*m*PPOX was a strong and selective inhibitor of Lip-HSL which only mildly alters Cut6 activity at a high molar excess. Thus, it is plausible that *in vivo* Lip-HSL proteins represent the primary targets of M*m*PPOX. MIC obtained with M*m*PPOX against *M. tuberculosis* or *M. bovis* BCG are slightly higher than those obtained with THL, which could be attributed to the ability of M*m*PPOX to inhibit a more restricted number of targets. In addition, culture conditions used here (aerobic, rich medium containing oleic acid) poorly induce expression of LipY [16] and presumably also other Lip-HSL members involved in the accumulation or catabolization of exogenous lipids. Thus, increased efficiency and lower MIC values are expected to occur when testing the inhibitor with mycobacteria growing in lipid-rich environment or with intra-cellular replicating mycobacteria. Furthermore, our data suggest that the Lip-HSL family fulfills essential metabolic and/or physiologic functions in *M. tuberculosis*.

Whether Lip-HSL proteins represent the only targets of M*m*PPOX *in vivo* remains however to be established. Chemical proteomic approaches involving tagged or grafted M*m*PPOX and incubation with whole mycobacterial cells will soon be performed to determine the selectivity spectrum of this compound. Finally, another important future issue emerging from this study is related to the impact of oxadiazolone compounds on the ability of mycobacteria to store and/or assimilate lipids during persistence and reactivation phases. THL was already investigated for its potency to prevent ILI catabolism in *M. smegmatis* [57], while this characteristic has not been investigated yet for M*m*PPOX. Experiments involving cellular and animal infection models are currently underway in our laboratory to address this important issue.

## Supporting Information

Table S1 Genes and physical properties of recombinant lipolytic enzymes.
(DOC)

Table S2 Expression and purification conditions of *M. tuberculosis* Lip-HSL proteins.
(DOC)

Table S3 Classification of genes encoding putative lipolytic enzymes found in the *M. tuberculosis* genome.
(DOC)

Table S4 PMF analyses of HSL family members and modifications after incubation with M*m*PPOX (*x*_I_ = 20).
(DOC)
