# Supplementary material for: MmPPOX Inhibits Mycobacterium tuberculosis Lipolytic Enzymes Belonging to the Hormone-Sensitive Lipase Family and Alters Mycobacterial Growth
Source: PLoS One. 2012 Sep 28;7(9):e46493. doi: 10.1371/journal.pone.0046493 (PMC3460867; doi:10.1371/journal.pone.0046493)
Supplement: Table S1 — Genes and physical properties of recombinant lipolytic enzymes. (DOC) [file pone.0046493.s001.doc]

**TABLE** **S1** Genes and physical properties of recombinant lipolytic enzymes.

| Gene | | | | | Putative protein*^a^* | | |
| --- | --- | --- | --- | --- | --- | --- | --- |
|  |  | ORF numbers | | | Molecular weight (Da) | Isoelectric point | Extinction coefficient (280 nm) |
| Protein | Cosmid/Bacmid | H37Rv*^b^* | BCG*^c^* | Identities*^d^* (%) |
| LipC | MTCY08D5.15/Bac329 | *Rv0220* | *BCG_0257* | 100 | 46113 | 9.9 | 1.43 |
| LipF | MTCY13E12.41/Bac233 | *Rv3487c* | *BCG_3551c* | 99 | 31104 | 7.0 | 0.91 |
| LipH | MTCY21B4.16c | *Rv1399c* | *BCG_1460c* | 99 | 36321 | 4.9 | 1.44 |
| LipI | MTCY21B4.17c/Bac255 | *Rv1400c* | *BCG_1461c* | 100 | 35727 | 5.2 | 1.42 |
| LipM | MTCY339.26c/Bac42 | *Rv2284* | *BCG_2299* | 100 | 46148 | 8.6 | 1.49 |
| LipN | MTCY349.17/Bac30 | *Rv2970c* | *BCG_2991c* | 100 | 41752 | 6.1 | 1.15 |
| LipO | MTCY493.28/Bac255 | *Rv1426c* | *BCG_1487c* | 100 | 47772 | 10.0 | 1.73 |
| LipQ | Genome | *Rv2485c* | *BCG_2503c* | 100 | 46924 | 8.8 | 1.47 |
| LipR | Bac48 | *Rv3084* | *BCG_3109* | 99 | 34291 | 9.5 | 0.87 |
| LipU | Bac174 | *Rv1076* | *BCG_1134* | 99 | 33455 | 6.1 | 1.06 |
| LipW | MTCY08D5.12c/Bac329 | *Rv0217c* | *BCG_0254c* | 99 | 33892 | 7.8 | 1.25 |
| LipY | MTCY164.08/Bac48 | *Rv3097c* | *BCG_3122c* | 99 | 47095 | 4.9 | 1.58 |

*^a^* Data were calculated using the ProtParam tool (<http://ca.expasy.org/tools/protparam.html>) with protein sequences including the His_6_-tag.

*^b^* Corresponding ORF numbers in *M. tuberculosis* H37Rv (<http://genolist.pasteur.fr/TubercuList/>)

*^c^* Corresponding ORF numbers in *M. bovis* BCG str. Pasteur 1173P_2_ (<http://genolist.pasteur.fr/BCGList/>)

*^d^* Amino acid sequence identities of *M. bovis* BCG as compared to *M. tuberculosis* H37Rv. Calculated using EMBOSS (<http://emboss.open-bio.org/>).
