# Supplementary material for: MmPPOX Inhibits Mycobacterium tuberculosis Lipolytic Enzymes Belonging to the Hormone-Sensitive Lipase Family and Alters Mycobacterial Growth
Source: PLoS One. 2012 Sep 28;7(9):e46493. doi: 10.1371/journal.pone.0046493 (PMC3460867; doi:10.1371/journal.pone.0046493)
Supplement: Table S2 — Expression and purification conditions of M. tuberculosis Lip-HSL proteins. (DOC) [file pone.0046493.s002.doc]

**TABLE S2** Expression and purification conditions of Lip-HSL proteins of *M. tuberculosis*.

|  |  |  |  |  | Ni^2+^-NTA | |  |
| --- | --- | --- | --- | --- | --- | --- | --- |
| Protein | Bacteria | Medium | Antibiotic*^a^* | T°C | Buffer A*^b^* | Imidazole*^c^* | Gel filtration |
| LipH | *E. coli* BL21 pLysS | TB | Amp | 17°C | 1 + urea 8 M | 50 mM | 2 |
| LipI | *E. coli* Rosetta pLysS | TB | Amp | 17°C | 1 + urea 8 M | 50 mM | 3 |
| LipN | *E. coli* Origami pLysS | TB | Amp | 17°C | 1 | 250 mM | 1 |
| LipC | *M. smegmatis* | 7H9 | Hyg | 37°C | 4 | 100 mM | 4 |
| LipF | *M. smegmatis* | 7H9 | Hyg | 37°C | 4 | 150 mM | 4 |
| LipM | *M. smegmatis* | 7H9 | Hyg | 37°C | 4 | 10 mM | 4 |
| LipO | *M. smegmatis* | 7H9 | Hyg | 37°C | 4 | 10 mM | 4 |
| LipQ | *M. smegmatis* | 7H9 | Hyg | 37°C | 4 | 10 mM | 4 |
| LipR | *M. smegmatis* | 7H9 | Hyg | 37°C | 4 | 250 mM | 4 |
| LipU | *M. smegmatis* | 7H9 | Hyg | 37°C | 4 | 150 mM | 4 |
| LipW | *M. smegmatis* | 7H9 | Hyg | 37°C | 4 | 50 mM | 4 |
| LipY | *M. smegmatis* | 7H9 | Hyg | 37°C | 5 | 250 mM | 5 |
| Cut6 | *E. coli* Chaperone | TB | Amp | 17°C | 5 | 250 mM | 5 |

*^a^* Antibiotics: Amp, ampicillin 100 µg/mL; Hyg, hygromicin 50 µg/mL

*^b^* Buffers list: 1, Phosphate buffer 50 mM NaCl 300 mM pH 8.0; 2, Tris-HCl 50 mM pH 7.0; 3, HEPES 10 mM NaCl 150 mM pH 7.5; 4, Tris-HCl 10 mM NaCl 300 mM pH 8.0; 5, Tris-HCl 10 mM NaCl 150 mM pH 8.0.

*^c^* Imidazole concentration needed to elute the protein.
