# Supplementary material for: MmPPOX Inhibits Mycobacterium tuberculosis Lipolytic Enzymes Belonging to the Hormone-Sensitive Lipase Family and Alters Mycobacterial Growth
Source: PLoS One. 2012 Sep 28;7(9):e46493. doi: 10.1371/journal.pone.0046493 (PMC3460867; doi:10.1371/journal.pone.0046493)
Supplement: Table S3 — Classification of genes encoding putative lipolytic enzymes found in the M. tuberculosis genome. (DOC) [file pone.0046493.s003.doc]

**TABLE S3** Classification of genes encoding putative lipolytic enzymes found in the *M. tuberculosis* genome.

| Related to | ORF number / gene name | Identity*^a^* (%) | Related publication |
| --- | --- | --- | --- |
| *Candida parapsilosis* lipase lip1 | *Rv1592*c | 26 |  |
| Human bile salt-dependent lipase | *Rv2045c* (*LipT*) | 25 |  |
| Human hormone-sensitive lipase | *LipC* | 14 |  |
|  | *LipF* | 22 |  |
|  | *LipH* | 22 |  |
|  | *LipI* | 25 | This work |
|  | *LipM* | 13 | This work |
|  | *LipN* | 21 | This work |
|  | *LipO* | 11 | This work |
|  | *LipQ* | 15 | This work |
|  | *LipR* | 18 | This work |
|  | *LipU* | 23 | This work |
|  | *LipW* | 22 | This work |
|  | *LipY* | 18 |  |
| *Fusarium solani* cutinase | *Cfp21* | 20 |  |
|  | *Cut1* | 16 |  |
|  | *Cut2* | 22 |  |
|  | *Cut3* | 18 |  |

**TABLE S3** Continued

| *Fusarium solani* cutinase | *Cut4* | 25 |  |
| --- | --- | --- | --- |
|  | *Cut5b* | 19 |  |
|  | *Cut6* | 16 |  |
| Mouse monoglyceride lipase | *Rv0183* | 35 |  |
|  | *Rv2715* | 12 |  |
|  | *Rv3203* (*LipV*) | 12 |  |
|  | *Rv3171c* | 23 |  |
| *Pseudomonas aeruginosa* phospholipase PLC-H | *Rv2349c* (*PLCC*) | 37 |  |
|  | *Rv2350c* (*PLCB*) | 37 |  |
|  | *Rv2351c* (*PLCA*) | 36 |  |
|  | *Rv1755c* (*PLCD*) | 38 |  |
| Others | *Rv0646c* (*LipG*) | - |  |
|  | *Rv0774c* | - |  |
|  | *Rv1192* | - |  |
|  | *Rv3338* | - |  |
|  | *Rv2797c* | - |  |
|  | *Rv3591c* | - |  |
|  | *Rv1683* | - |  |

*^a^*Identities were calculated using EMBOSS (<http://emboss.open-bio.org/>).

### References

1. Raman K, Yeturu K, Chandra N (2008) targetTB: A target identification pipeline for *Mycobacterium tuberculosis* through an interactome, reactome and genome-scale structural analysis. Bmc Systems Biology 2.

2. Camus JC, Pryor MJ, Medigue C, Cole ST (2002) Re-annotation of the genome sequence of *Mycobacterium tuberculosis* H37Rv. Microbiology-Sgm 148: 2967-2973.

3. Shen G, Singh K, Chandra D, Serveau-Avesque C, Maurin D, et al. (2011) LipC (Rv0220) is an immunogenic cell-surface esterase of *Mycobacterium tuberculosis*. Infect Immun 80: 243-253.

4. Zhang M, Wang JD, Li ZF, Xie J, Yang YP, et al. (2005) Expression and characterization of the carboxyl esterase Rv3487c from *Mycobacterium tuberculosis*. Protein Expression and Purification 42: 59-66.

5. Canaan S, Maurin D, Chahinian H, Pouilly B, Durousseau C, et al. (2004) Expression and characterization of the protein Rv1399c from *Mycobacterium tuberculosis* - A novel carboxyl esterase structurally related to the HSL family. European Journal of Biochemistry 271: 3953-3961.

6. Deb C, Daniel J, Sirakova TD, Abomoelak B, Dubey VS, et al. (2006) A novel lipase belonging to the hormone-sensitive lipase family induced under starvation to utilize stored triacylglycerol in *Mycobacterium tuberculosis*. J Biol Chem 281: 3866-3875.

7. Mishra KC, De Chastellier C, Narayana Y, Bifani P, Brown AK, et al. (2008) Functional role of the PE domain and immunogenicity of the *Mycobacterium tuberculosis* triacylglycerol hydrolase LipY. Infection and Immunity 76: 127-140.

8. Schué M, Maurin D, Dhouib R, N'Goma JCB, Delorme V, et al. (2010) Two cutinase-like proteins secreted by *Mycobacterium tuberculosis* show very different lipolytic activities reflecting their physiological function. FASEB J 24: 1893-1903.

9. West NP, Chow FM, Randall EJ, Wu J, Chen J, et al. (2009) Cutinase-like proteins of *Mycobacterium tuberculosis*: characterization of their variable enzymatic functions and active site identification. Faseb J 23: 1694-1704.

10. Meniche X, Labarre C, de Sousa-d'Auria C, Huc E, Laval F, et al. (2009) Identification of a Stress-Induced Factor of Corynebacterineae That Is Involved in the Regulation of the Outer Membrane Lipid Composition. Journal of Bacteriology 191: 7323-7332.

11. Parker SK, Barkley RM, Rino JG, Vasil ML (2009) *Mycobacterium tuberculosis* Rv3802c encodes a phospholipase/thioesterase and is inhibited by the antimycobacterial agent tetrahydrolipstatin. PLoS ONE 4: e4281.

12. Côtes K, Dhouib R, Douchet I, Chahinian H, de Caro A, et al. (2007) Characterization of an exported monoglyceride lipase from *Mycobacterium tuberculosis* possibly involved in the metabolism of host cell membrane lipids. Biochem J 408: 417-427.

13. Dhouib R, Laval F, Carriere F, Daffé M, Canaan S (2010) A Monoacylglycerol Lipase from *Mycobacterium smegmatis* Involved in Bacterial Cell Interaction. Journal of Bacteriology 192: 4776-4785.

14. N'Goma JCB, Schué M, Carrière F, Geerlof A, Canaan S (2010) Evidence for the cytotoxic effects of *Mycobacterium tuberculosis* phospholipase C towards macrophages. Biochimica Et Biophysica Acta-Molecular and Cell Biology of Lipids 1801: 1305-1313.

15. Singh G, Jadeja D, Kaur J (2010) Lipid hydrolizing enzymes in virulence: *Mycobacterium tuberculosis* as a model system. Critical Reviews in Microbiology 36: 259-269.

16. Gu S, Chen J, Dobos KM, Bradbury EM, Belisle JT, et al. (2003) Comprehensive proteomic profiling of the membrane constituents of a *Mycobacterium tuberculosis* strain. Molecular & Cellular Proteomics 2: 1284-1296.

17. Low KL, Shui GH, Natter K, Yeo WK, Kohlwein SD, et al. (2010) Lipid Droplet-associated Proteins Are Involved in the Biosynthesis and Hydrolysis of Triacylglycerol in *Mycobacterium bovis* Bacillus Calmette-Guerin. Journal of Biological Chemistry 285: 21662-21670.
