# Supplementary material for: MmPPOX Inhibits Mycobacterium tuberculosis Lipolytic Enzymes Belonging to the Hormone-Sensitive Lipase Family and Alters Mycobacterial Growth
Source: PLoS One. 2012 Sep 28;7(9):e46493. doi: 10.1371/journal.pone.0046493 (PMC3460867; doi:10.1371/journal.pone.0046493)
Supplement: Table S4 — PMF analyses of HSL family members and modifications after incubation with MmPPOX (x I = 20). (DOC) [file pone.0046493.s004.doc]

**TABLE S4** PMF analyses of HSL family members and modifications after incubation with M*m*PPOX (*x*_I_ = 20).

|  |  |  |  | Theoretical masses*^b^* (Da) | Experimental masses*^c^* (Da) | | |
| --- | --- | --- | --- | --- | --- | --- | --- |
| Protein | Protease used | Matching peptides*^a^* | Sequence coverage (%) | Unmodified catalytic peptide | Unmodified catalytic peptide*^d^* | Modified catalytic peptide | Modification mass*^e^* |
| LipC | Trypsin | 12 | 41 | 4762.305 | ND | - | - |
|  | Chymotrypsin | 22 | 50 | 1055.530 | ND | - | - |
| LipF | Trypsin | 18 | 64 | 2589.378 | 2589.393 | ND | - |
| LipH | Trypsin | 15 | 47 | 1972.028 | 1972.022 | 2256.113 | +284.085 |
|  |  |  |  | 1988.023 | 1988.016 | 2272.114 | +284.091 |
| LipI | Trypsin | 15 | 65 | 1740.960 | 1741.279 | 2025.401 | +284.441 |
| LipN | Trypsin | 27 | 87 | 2001.018 | 2000.996 | 2285.041 | +284.023 |
|  |  |  |  | 2244.140 | ND | - | - |
| LipR | Trypsin | 8 | 18 | 2059.121 | ND | - | - |
|  | Chymotrypsin | 18 | 36 | 1458.743 | 1458.813 | ND | - |
| LipU | Trypsin | 24 | 80 | 2788.488 | 2788.475 | ND | - |
| LipW | Trypsin | 15 | 57 | 1780.044 | 1780.071 | ND | - |
| LipY | Chymotrypsin | 35 | 60 | 2310.168 | 2310.143 | ND | - |
|  |  |  |  | 2494.289 | 2494.283 | ND | - |

*^a^* Matching peptides were identified using MASCOT^TM^ search engine against NCBI database.

*^b^* Theoretical monoisotopic values ([M+H]^+^) were measured using the BioTools^TM^ software (Bruker, Daltonik, Deutchland), assuming no miss cleavage for trypsin and one miss cleavage maximum for chymotrypsin, Cysteines treated with iodoacetamide and Methionines optionally oxidized (+16 Da per oxidized Methionines).

*^c^* Experimentally measured monoisotopic [M+H]^+^.

*^d^* Measured from incubation without M*m*PPOX.

*^e^* Calculated using the theoretical mass of the unmodified peptide as reference.

ND: not detected.
